# Factors Affecting Training and Physical Performance in Recreational Endurance Runners

**DOI:** 10.3390/sports8030035

**Published:** 2020-03-15

**Authors:** Daniel Boullosa, Jonathan Esteve-Lanao, Arturo Casado, Leonardo A. Peyré-Tartaruga, Rodrigo Gomes da Rosa, Juan Del Coso

**Affiliations:** 1 Graduate Program in Movement Sciences, Federal University of Mato Grosso do Sul, Campo Grande, Mato Grosso do Sul 79070-900, Brazil; 2 All in Your Mind Training System™, Mérida, Yucatán 97134, Mexico; jonathan.esteve@allinyourmind.es; 3 Faculty of Health Sciences, Isabel I de Castilla International University, Burgos, 09003 Castilla y León, Spain; arturocasado1500@gmail.com; 4 Exercise Research Laboratory, Universidade Federal do Rio Grande do Sul, Porto Alegre, Rio Grande do Sul 90690-200, Brazil; leotartaruga@gmail.com (L.A.P.-T.); rodrigogomesdarosa@gmail.com (R.G.d.R.); 5 Centre for Sport Studies, Rey Juan Carlos University, Fuenlabrada, 28943 Madrid, Spain; juan.delcoso@urjc.es

**Keywords:** endurance exercise, sports performance, sports training, running technique, exercise intensity, amateur runner

## Abstract

Endurance running has become an immensely popular sporting activity, with millions of recreational runners around the world. Despite the great popularity of endurance running as a recreational activity during leisure time, there is no consensus on the best practice for recreational runners to effectively train to reach their individual objectives and improve physical performance in a healthy manner. Moreover, there are lots of anecdotal data without scientific support, while most scientific evidence on endurance running was developed from studies observing both recreational and professional athletes of different levels. Further, the transference of all this information to only recreational runners is difficult due to differences in the genetic predisposition for endurance running, the time available for training, and physical, psychological, and physiological characteristics. Therefore, the aim of this review is to present a selection of scientific evidence regarding endurance running to provide training guidelines to be used by recreational runners and their coaches. The review will focus on some key aspects of the training process, such as periodization, training methods and monitoring, performance prediction, running technique, and prevention and management of injuries associated with endurance running.

## 1. Introduction

Endurance running has been suggested to be a biological trait associated with the survival of our species [1,2]. In fact, our species could be considered one of the best examples of natural endurance athletes, with neurobiological mechanisms reinforcing the search for habitual aerobic exercise, particularly running [3]. Therefore, it is easy to understand why millions of people compete every weekend in road endurance running races of variable distances, from 5 km to marathon races, around the world [4,5]. Currently, a great number of recreational runners train every day in different places. The success of this recreational sport might be attributed to its simplicity and accessibility while favoring health development [6], and the social interactions and leisure experiences that it lends to most people, regardless of level or age.

Despite its great popularity, there is no consensus on the best practice when looking for sustained or improved endurance running performance in recreational running while maintaining or enhancing health status. However, there are a plethora of anecdotal guidelines without robust scientific support, including the use of nonvalidated algorithms (e.g., Trainingpeaks™) for training monitoring, the “10% rule” for weekly training load increments, or the use of different types of shoes to reduce injury rates. In fact, it is common to see recreational runners imitating training practices similar to those of professional athletes, including completion of high weekly mileage (e.g., > 70 km), which may be behind the high prevalence of some health-related problems [7]. Further, as current scientific evidence on endurance running was developed from studies observing recreational and professional athletes of very different levels (from untrained to elite), it is unclear how recreational runners can effectively use all the available information. Furthermore, the definition of a recreational runner is very wide, since we could include in this category any runner who trains and competes regularly during leisure time independently of the performance level (from novice to well-trained athletes) and specific objectives (e.g., enjoyment, health, competition, etc.). However, in contrast to professional athletes, recreational runners have limited schedules to train, which often are adapted to other daily living activities and duties. Moreover, although some ex-professional athletes exist in the recreational runner category, it is expected that most recreational runners do not possess the genetic advantages and the physical, physiological, and psychological traits of professional endurance runners. 

Therefore, the aim of this review is to present a selection of scientific evidence regarding recreational runners to identify best practices and to present a starting point for further studies regarding this population. This narrative review will focus, based on our practical experience, on some key aspects, such as periodization, training methods and monitoring, performance prediction, running technique, and prevention and management of injuries associated with endurance running. The information provided here may be useful for recreational runners and their coaches to optimize the process of endurance running training in order to look for better performances in a healthy manner. However, adapting all of this information should be performed on an individual basis while assuming that some specific topics could be missing.

## 2. Training Characteristics of Recreational Endurance Runners

The raining characteristics of recreational runners comprise several relevant topics. Therefore, this section reviews information regarding running training methods, strength training for endurance running, training intensity distribution, and training periodization. 

### 2.1. Running Training Methods

Training sessions that include continuous exercise performed at both low and high intensity levels and interval training, including sessions of variable intensity, represent the training methods most often used to improve performance in endurance competitive events [8]. Evidence suggests that several forms of training with single use and combination of continuous and interval training methods are effective to improve performance in recreational endurance runners, especially in investigations where study samples were composed of athletes with less comprehensive training backgrounds. Several studies involving the use of high-intensity interval training (HIIT) observed performance improvements after interventions from four to ten weeks [9,10,11,12,13,14,15,16,17,18,19]. Studies combining the use of continuous submaximal running (CT) and HIIT found further improvements in performance [10,14,16,18]. Of note, some previous studies used sprint interval training (SIT, all-out efforts lasting 20–30 s with long resting periods of 3–5 min), which also generated improvements in performance in 3 and 10 km competitions [10,15,18]. In addition, studies investigating aerobic HIIT (2–4 min at an intensity ≤ 100% of the velocity associated to maximum oxygen consumption [vVO_2_max]) also found improvements in performance in running competitions from 1.5 to 10 km [10,11,12,13,14,15,16], while short intervals of HIIT (lasting 20–60 s at a higher intensity than the vVO_2_max) also exhibited improvements in performance from 1.5 to 10 km [9,12,13,17,18]. All of these previous studies suggested that different forms of HIIT, such as SIT, aerobic HIIT, or HIIT with short intervals, could be implemented in order to improve the performance of recreational runners. However, a combination of several methods might be the best option to improve adaptation to endurance running training. 

Aerobic HIIT [11,12,14,15,19] and short-interval HIIT [9,11,12,16] were deemed as effective exercise modalities to VO_2_max. However, Denadai et al. [13] did not find improvements in VO_2_max after aerobic HIIT in well-trained runners, indicating that short-interval HIIT at very high intensity might be necessary to produce any significant effect in well-trained athletes. On the other hand, aerobic HIIT was found to improve the velocity associated with VO_2_max (vVO2max; [11,14,15]), thereby implicating improvements in running performance even without the presence of higher VO_2_max values. Running economy (RE), or the energetic cost of running at a given running speed [20], was found to improve after aerobic HIIT [13,15,19] and SIT-based programs [10,17]. The lack of RE improvement after a short HIIT intervention [12] suggests that improvements in RE are not only related to the type of HIIT used but also to the total volume completed. In this regard, RE improvements were related to improvements of muscle oxidative capacity, as suggested by Denadai et al. [13]. Interestingly, studies comparing training programs with HIIT and CT showed greater improvements in VO_2_max [18] and vVO_2_max [19] when using HIIT, and in RE when using CT [19].

Well-structured intervention studies utilizing other types of running methods are scarce in literature (e.g., running on sand, fartlek, etc.), therefore comparing these and other methods through examination of both physiological and performance adaptations is recommended in future studies. Therefore, a combination of methods, including one to two HIIT sessions per week plus more sessions with moderate- and low-intensity CT, is generally recommended to improve performance in recreational runners in a healthy manner.

### 2.2. Strength Training

In addition to running, strength training (ST) was found to improve muscular strength and RE in runners of very different levels [21,22,23], and performance in previously trained runners [23]. Thus, RE improvements were observed following different types of ST, such as resistance training [24,25,26,27] and plyometric training [28]. In addition, ST might be also effective to improve the VO_2_max of novice recreational runners after 6 to 14 weeks of training [24,28,29,30], but this finding was not observed in well-trained runners. One study [31] found that neither physiological nor performance benefits after the use of ST by recreational marathon runners. In this regard, it was suggested that the lack of improvement in RE may be due to the small sample size used and the short intervention completed [31]. However, the lack of physiological and performance improvements generated by ST may also be explained by the reduction in muscle size that happens after a marathon training period in recreational runners, which may be indicative of an adaptation to reduce oxygen consumption at submaximal intensities [32]. Given that ST is expected to be related to some hypertrophic response [33], ST could impair marathon performance, despite the fact that the hypertrophic effect is attenuated when both ST and endurance training are concurrently performed [34]. In any case, Blagrove and colleagues [21] suggested the use of free-weight multi-joint exercises to improve running performance. Although there is a lack of knowledge regarding the optimal volume and intensity of ST for endurance running performance [21], the inclusion of ST in the training regime of recreational runners might be recommended, even for marathoners. However, ST should be always performed with caution, while looking for the minimum dose to allow significant neuromuscular adaptation.

### 2.3. Training Intensity Distribution

According to Seiler and Kjerland, three intensity zones can be delimited by capillary blood lactate responses to steady state continuous running: Zone 1, the low lactate metabolic phase; Zone 2, the lactate accommodation phase, where blood lactate concentration is higher but production and removal rates are in equilibrium; and Zone 3, the lactate accumulation uneven phase, where blood lactate production exceeds the maximum clearance rate [35]. Polarized training intensity distribution (TID) [35,36,37] is conducted with a significant percentage of time in Zone 1 (75%–80%) and Zone 3 (15%–20%) but with little or no time in Zone 2. TID was suggested to be more efficient than other intensity distributions because it is linked to the physical activity pattern of our ancestors [38]. In contrast, Holmberg proposed the traditional “pyramidal” TID, in which most of the training time is conducted in Zone 1 (70%–80%), with the remaining 20%–30% between Zones 2 and 3 [39]. Nonetheless, the literature regarding TID in recreational runners is scarce, therefore it is difficult to ascertain what models might be more beneficial for these runners. Muñoz et al. found that, despite the reduced volume conducted by recreational runners, a polarized model improved their 10 km performance to a greater extent than a threshold model, in which most of the training volume was conducted in Zone 2 [40]. More recently, it was observed that mesocycles with different TIDs (polarized vs. HIIT vs. low intensity) resulted in similar improvements and physiological adaptations [41]. However, the low-intensity group, in which most of the training was conducted in Zone 1, induced improvement in RE, which was not observed in the other groups [41]. Finally, another recent study found that polarized TID improved both RE and performance in ultra-endurance runners [42]. The pyramidal and polarized models might be the most recommended forms of training intensity distribution for recreational runners, with different considerations depending on competitive distance, time available to train, and time of the season. Meanwhile, “high volume” or “high intensity” approaches seem to fail to reach optimal results, and only very low fitness runners seem to benefit from any training approach in the short-term. 

### 2.4. Training Periodization

Periodization refers to subdivision of a training plan into shorter periods by means of manipulating volume and intensity over a season. Overall, periodization may be categorized into two main models, namely, block periodization and traditional periodization [43]. Block periodization refers to the subdivision of an annual plan into shorter periods (blocks) of highly specific and concentrated workloads, whereas traditional or linear periodization involves different cycles in which different contents are present, but with volume decreasing proportionally to the increase of intensity throughout the season [43]. An alternative traditional periodization is reverse linear periodization, in which the intensity is high and the volume is low in the initial stages of training with the intensity subsequently decreasing and the volume increasing until the end of the training cycle. Research on training periodization in recreational runners is very limited. Bradbury et al. found that linear and reverse linear periodization generated greater improvements in endurance performance, RE, and VO_2_max in recreational runners than nonperiodized progression of training load [44]. Further, when considering previous studies including the use of HIIT and SIT in recreational runners, it may be suggested that, during the training period, recreational runners should adopt a linear periodization when using HIIT (from high to low volume and from low to high intensity) and a reverse linear periodization when using SIT in order to increase performance [10,14,16,18,45], following the principle of progressive overload. In addition, a general recommendation from our practical experience would be that either block or linear periodization may fit better with short or moderate distances (21 km or shorter), while performance in marathons and longer events would probably benefit from reverse periodization. Novice, less experienced runners would also benefit from reverse periodization for 21 km or even 10 km events, in order to be exposed more gradually to peak volume. Further studies are needed to appropriately test the validity of these suggestions.

All these findings suggest that both HIIT and ST should be implemented in the training regime of recreational runners in order to improve performance and its determining factors, such as RE and VO_2_max. In addition, recreational runners should adopt a polarized TID, although other models may also be beneficial; however, an important aspect to be considered is that most training time (≥75%) should be completed in Zone 1 for better results. Furthermore, runners should conduct periodized training in order to maximize improvements in performance rather than using nonperiodized training. However, the limited amount of research regarding both TID and training periodization in recreational runners must be acknowledged, therefore, more research regarding these issues would improve the understanding of the underpinning mechanisms involved in the responses of recreational runners to training. In this regard, the time completed in Z1 could be more important than intensity polarization per se, and both testing sessions and competitions should be also included in TID calculations [46]. In addition, the minimum running volume required to improve running performance in recreational runners [47], including the interactions between TID, running, and ST methods [48] and the significant influence of incidental physical activity on running training adaptations [49] still require identification.

## 3. Training Monitoring

Recreational runners can monitor their progress through several traditional and modern parameters obtained from training and testing sessions, or competitions. Namely, traditional laboratory evaluations aiming to identify changes in gold standard physiological measures, such as VO_2_max, anaerobic threshold (AT), and RE, can be monitored in recreational runners alongside professional runners [50]. However, the assessment of these maximal and submaximal variables is not accessible to all runners in terms of cost or availability. Thus, other low-cost and simple evaluations could be used, especially in the field. From these tests, incremental field-testing for identification of maximum aerobic speed (MAS) could be considered the most practical, as MAS integrates both VO_2_max and RE into a single parameter and was used regarding endurance running performance from middle- to long-distance races [51]. In addition, incremental-intensity field-testing could serve to identify the true maximum heart rate (HRmax) [52] and the AT based on HR measures [53], thereby allowing both monitoring of running capacity and training prescription based on these parameters. In this regard, the five minute running test (T5) could be recommended as a simple and valid alternative for MAS determination in the field [54], although MAS identified using T5 presents greater error and lower reproducibility when compared to MAS recorded using an incremental test [55], probably because of the influence of pacing strategies in T5. 

Other field-testing evaluations include the calculation of critical speed (CS; i.e., the highest sustainable running speed that can be maintained without a continual rise in VO_2_) from a number of maximum time trials [56], the identification of HR-running velocity associations at submaximal intensities [57], and AT determination with the changes produced in blood lactate concentration with intensity [58,59]. However, CS ideally requires maximal testing on different days, therefore it does not represent an appealing option for some recreational runners, while the blood lactate measures required for AT testing may also represent a barrier in some cases. In contrast, methods identifying the relationship between submaximal constant velocity bouts and HR responses could be a simple and valid alternative [60] when controlling factors affecting HR monitoring, such as dehydration [61]. In addition, the combination of constant velocity and incremental tests in the field was also suggested to be valid in identifying the anaerobic threshold along with the MAS [62]. Of note, field running testing could be more difficult to apply due to the constraints associated with field and weather conditions. However, field testing may present greater validity for training prescriptions when compared to laboratory-based testing on a treadmill [63]. Therefore, runners and coaches should identify the tests that better suit the needs and possibilities for monitoring targeted physiological adaptations to training over the season for specific objectives. Furthermore, although the best performance test is to compete in the same distance to compare performances and physiological responses between races, it is noteworthy that differences in surfaces, profiles, and weather conditions between races challenge such comparisons and therefore complicate appropriate diagnoses of competitive outcomes. Meanwhile, these limitations should be also considered for daily quantification of TID via methods based on HR or speeds associated with physiological thresholds, which are not interchangeable. Alternatively, the use of a percentage of goal race pace could be also recommended for TID monitoring [64].

Another valid approach may include frequent monitoring of a typical training session with comparison of both external (e.g., running velocity) and internal (e.g., HR) load parameters [65]. Thus, if the athlete ran faster a given distance with the same HR, or reduced their HR when running at the same velocity, this would show a positive adaptation with a lower internal load for a given external load [66]. Comparing internal and external load parameters in real-time is currently very easy with the use of wearable technology that simultaneously records various parameters, including HR, velocity with GPS technology, kinematics (i.e., stride rate and length) via accelerometry, altitude, and temperature, among others [67]. However, there is no evidence demonstrating whether wearable technology is more valid and efficient than simple monitoring tools, such as session rating of perceived exertion (sRPE) and training impulse (TRIMP = sRPE × session time), which were demonstrated to be valid for discriminating between short- and long-term adaptations [65]. This is an important consideration, as load indices such as monotony (i.e., mean weekly sRPE/standard deviation) and strain (i.e., weekly sRPE × monotony) could be very helpful to avoid overreaching and overtraining, requiring only the recording of this simple information on a daily basis [68].

Finally, over the last decade, a number of studies pointed out the validity and practicality of heart rate variability (HRV) for training monitoring in recreational runners [60,69,70]. HRV is the variation of HR over time, as calculated using different indices, and represents a measurement of autonomic nervous system activity, which correlates very well with psycho-physiological adaptations, stress tolerance, and the aerobic status of runners [71]. Thus, different studies found that grater vagal modulations are related to better training response, adaptation, and recovery in both low- and high-intensity training [69,72]. Moreover, several studies showed how training-load guided by HRV monitoring was more efficient than traditional periodization for training adaptations in both males [70] and females [73] when identifying greater-stress days, thereby reducing the runners’ potential for adaptation and requiring a lowered training load. While some HR monitors offer this monitoring tool, this approach is currently easier to implement with different easy-to-use apps (e.g., HRV4training; [74]), which integrate HRV measures with other load and psychometric measures.

There are several monitoring tools with varying levels of complexity and validity from which appropriate programs can be selected for each scenario and purposes. However, as the superiority of any method has not been demonstrated over others with respect to the validity and sensibility for training monitoring, the preference and accessibility of runners and their coaches to these methods may be the main criteria for selecting the most appropriate parameters in every scenario. In this regard, we encourage the use of several methods to simultaneously monitor the external and internal load parameters (e.g., sRPE + HRV + kinematics) to better assist with training decisions on a daily basis, while programming testing sessions regularly to objectively identify the targeted improvements of physiological and performance parameters in well-standardized conditions.

## 4. Performance Predictions

Since many runners aim to improve their personal best times, it is of interest to predict endurance performance during the training process. While both procedures are directly linked, performance prediction should not be confounded with training monitoring, as the latter focuses on the factors associated with improving the former. The most usual and intuitive way to predict performance is to theoretically calculate race time by using the best-time in races of different distances. This is possible as some equivalence is expected between times over different distances [75]. As an example, these formulas multiply (or divide) the time employed to cover a given distance to obtain the prediction of the desired distance (i.e., marathon time = 10 km time × 4.76). Following this method, it is assumed that the distance equivalence corresponds to a time ratio for one distance to the next, plus extra time due to the effect of fatigue [76].

Apart from many limitations (state of preparedness along time, individual characteristics over some distances, etc.), this method is considered the most accurate to predict running performance [77,78,79,80]. However, these predictions do not provide solutions for the training process, as they do not reveal what physiological parameters are involved in achieving that performance. Through this approach, it would be only intuitive to consider whether the runner is “too slow or too resistant”. In addition, the completion of one maximal effort to estimate another distance performance may be inconvenient for most recreational runners.

To approach the necessity of some physiological information, Mercier et al. provided nomogram-related parameters, such as VO_2_max and metabolic thresholds, in order to predict times over a whole range of distances [81]. This approach was validated over different distances according to sex in several studies from Bosquet’s research group [75,82,83]. This nomogram represents a great tool that integrates different predictions that can be individually determined. Although different physiological parameters were included in different prediction models, the most important performance-related parameter is the peak velocity achieved at the end of a treadmill-graded test [78,84,85,86], followed by the vVO_2_max [87,88,89,90] and the velocity associated with the second metabolic threshold [91,92,93,94,95]. Although the factors determining endurance running performance could be independent predictors, an interaction among them was also observed [96]. Moreover, different regression models also included anthropometric and training parameters [91,94,97,98,99,100], while others used information from anthropometry, physiological parameters, and time over shorter distances [101]. 

Another interesting concept is the prediction of the rate of improvement over a number of years. In this regard, Péronnet proposed a number of variables to improve the model, including previous experience, performance times during the first systematic training season, performance time evolution and total training load over the years, previous experience, and willingness to push oneself through the training process [102].

More specifically for recreational marathon runners, Larumbe et al. recently added the rate of social support as a key element surrounding pre-race performance state, suggesting that emotional or social variables could also influence performance [103]. More recently, another approach considered in-season predictions using different equations over the training macrocycle to improve accuracy as the race day drew closer [104]. Contrary to other cross-sectional studies, these equations highlighted the validity and practicality of the velocities associated with the aerobic and anaerobic thresholds to predict marathon running performance while using these two fundamental training parameters.

The best way to predict recreational runners’ racing times may be to record individual performances over shorter distances and to conduct assessments close to the race pace or to the closer physiological intensity (i.e., lactate threshold). For instance, using formulas from a shorter distance while complementing this information with the evaluation of the expected steady state pace during the event (i.e., capillary blood lactate measurements) could be enough to predict running performance. From these predictions, coaches and runners should select a given method and apply it systematically, including not only repeating procedures and ambient conditions, but also considering the training stage of the individual at the moment of evaluation. These considerations, which are important for more accurate individual predictions, are not considered when developing predictive models based on big datasets [105].

## 5. Running Technique

Running technique (RT) could be defined as the automated arrangement of segmental movements to achieve optimal integrative responses during running [106]. RT can be analyzed by biomechanical methods and can be affected by intrinsic and extrinsic factors [107]. The intrinsic factors of RT include kinematic, kinetic, and neuromuscular parameters, while extrinsic factors include the shoe–surface interaction, with footwear, orthotics, and the running surface among other aspects [107]. The analyses provided by biomechanics from these different factors are important for understanding the performance or injury risk of different RT.

Since RT is a determining factor of RE, the modification of RT is one of the most important objectives for recreational runners [108], as it is expected that changes in RT induce changes in performance [109], or even lower the risk of injury [110]. The interventions available for RT improvement may be divided into direct and indirect interventions, where direct interventions involve the enhancement of specific running patterns using perceptual approaches, such as verbal and visual feedback [111,112], monitoring of tension in arms and shoulders [113], athletic skills [114,115,116], and habituation to different running patterns [117,118]; and indirect interventions are those designed to improve conditioning capacities, such as core stability, strength, and balance, that ultimately may influence the RT but without any direct intervention from factors associated with running patterns. 

Contrary to common belief, the search for a “one-fits-all” RT is not accompanied by improvement in performance or RE. In this regard, the recommendation of a global RT is not effective nor advisable. This is the case of the Pose® method, which has become a popular intervention for the modification of RT. Briefly, this method proposes running with a forward trunk lean, a more flexed position at touch-down, a mid-foot striking style, and a low range of arm motion [119]. A previous study demonstrated that adopting this particular RT did not result in changes in specific biomechanical factors associated with injury risk, nor in running performance [120]. Also, RE deteriorated when RT was altered with this method by reducing stride length and vertical oscillations [121]. In another recent study using the Pose® method for gait retraining in recreational runners, it was demonstrated that injury-related biomechanical markers and trunk kinematics remained similar after the training period [122]. Thus, direct interventions pursuing a determined RT did not show positive alterations to RE, nor to performance variables in runners.

On the other hand, many studies demonstrated that strength and explosive training programs are capable of improving RE and performance mediated by RT improvement, such as contact time reduction [123,124,125]. Maximum strength and explosive training programs may increase the net excitation of motoneurons, thus resulting in better RE [123]. While the long-term adjustments of RT are still unclear, recent evidence also suggested that isometric strength training could increase tendon–aponeurosis stiffness, thus resulting in enhanced RE [25]. Similarly, Pilates® training induced improvements in RE and 5 km times in recreational runners [126]. These modifications may be associated with enhanced muscle activation patterns during running.

Previous observational studies tested the hypothesis of an ideal RT related to RE and performance. Although there are inconsistent findings and limited understanding of the biomechanical factors related to optimal RT, the strongest direct links with RE are running biomechanics during the contact phase, particularly those during propulsion [107]. It was suggested that the runner’s experience is associated with RT [108], however the runner’s experience does not appear to significantly influence running mechanics [127]. In contrast, using the spring-mass model to understand the determining factors of RT, a recent study observed that faster recreational runners unveiled more “elastic” responses than slower runners [128]. The effective aerial time, the elastic system frequency, and the vertical stiffness were greater and the effective contact time was shorter in faster runners. These key parameters for RT optimization were also associated with greater running economy [128]. This evidence is linked to evidence demonstrating that aged people show systematic alterations in RT with reduced strength and power, therefore decreasing their ability to store elastic energy in muscle–tendon units [129,130], while also reducing stride length and increasing stride frequency in comparison with young adults.

Some simple RT changes can be induced using sound or visual feedback methods to modify stride frequency and length. Thus, a protocol for increasing 10% the preferred stride frequency, thus also reducing 10% of the preferred stride length, induced decreases in the peak hip adduction angle and vertical loading rates, which are parameters that were associated with iliotibial band syndrome risk [131,132]. In addition, when evaluating recreational runners while running at above the preferred step frequency, reduction of hip- and knee-joint loading during running was observed [133,134]. However, some caution should be taken when changing stride parameters to lower injury risk, as these modifications were related to negative changes in RE and performance [135,136]. Similarly, acute alterations in RT are controversial because their effectiveness to produce benefits are not well supported by current evidence. 

Another interesting topic that was recently investigated following the concerns of both coaches and runners is the effect of forefoot (FFS) and rearfoot strike (RFS) changes on RT, injury risk, and performance. Thus, when evaluating a group of runners using both strike patterns, no mechanical advantages of FFS vs. RFS runners were evident [137]. RFS runners switching to FFS may reduce their injury risk due to the altered distribution of loading between joints, however this should be weighed against possible performance decrements [137,138]. In another recent study, the retraining from RFS to FFS did not affect running economy, but did reduce running-related patellofemoral pain after one month of training [139]. Therefore, the objectives and the costs of altering the strike pattern in terms of injury risk and performance changes must be considered before any intervention. Also, expert opinion [110] and meta-analysis [140] confirmed the limited evidence that supported the efficacy of the transition from the RFS to the FFS technique. 

In summary, a specific foot-strike pattern does not reduce joint contact forces and the recommendations of changing RT should only be based on individual analysis by experts. Evaluation methods based on optimality are interesting for RT analysis; parameters that assess RT at an organismal level may show a clearer mechanistic model to explain the role of RT on performance in recreational runners. Models including integrative parameters, such as elastic bouncing and metabolic economy, have great potential for this purpose. Of note, optimal stride frequency and length (stride frequency and length where the metabolic cost is lowest) are normally found at freely chosen conditions [135,141,142]. Determining these optimal parameters at the individual level seems to be the better way to analyze RT in recreational runners. 

## 6. Factors Associated with Running-Related Injuries

Endurance running is very challenging for muscle and connective tissues because it is a weight-bearing activity involving stretch–shortening cycles repeated over time [143]. Longer running activities entail greater exercise time, thus, a concomitant higher number of ground impacts increase risk of overuse injuries. However, shorter endurance running activities are typically covered at faster paces, which increase peak vertical and horizontal forces [144], thereby leading to sudden-onset overload injuries. Thus, all types of endurance running activities are subject to a certain risk of injury in the lower limbs [145]. In addition, extensive evidence indicates that several intrinsic and extrinsic factors might contribute to increased risk of injury in recreational runners. Because sustaining a running-related injury withdraws the benefits of continuous running practice, the study of the factors that lead to injury might be essential to reduce the probability of sustaining an injury in this population. 

Previous literature consistently revealed that running-related injuries affect most runners. However, the exact prevalence of running-related injuries in recreational endurance runners is difficult to ascertain, because studies have been inconsistent when reporting results of injury incidence (e.g., the number of injuries per 1000 km of running or per 1000 h of running, number of injuries in a population of endurance runners, percentage of injured runners during a set period of time [146]). The overall incidence of lower extremity injuries might vary from ~20% to ~80% of the runners involved in endurance running training or competition during one year [145]. If the incidence is calculated according to running time exposure, the incidence reported in the literature varies from 2.5 to 33.0 injuries per 1000 h [146,147]. Overall, injury incidence is greater in marathoners than in other runners focused on shorter endurance competitions [148], while injury incidence increases with the competitive distance (e.g., ultra-marathoners [149]). Injuries sustained during training are more common than injuries sustained during competition, but the incidence of injuries during training increases in the month prior to competing in a race [150], likely due to the increased training intensity as a consequence of tapering strategies. More importantly, novice runners have a higher injury risk than more experienced runners [146,151]. Therefore, injury risk may be greater for those who have recently started to run, those with sudden increments in either training load or intensity, and those training for longer distances, as these people need higher running volumes to prepare for races. Specifically for novice runners, one interesting approach to reduce muscle strain and discomfort during running with no effect in final times is the use of a walk/run strategy [152]. However, the effectiveness of this approach for injury risk reduction is yet to be determined. 

The most important intrinsic risk factor for endurance running injury is the existence of a previous injury, indicating that special attention should be taken to avoid recurrent injuries in endurance runners [153,154]. Although the mechanism that induces an injury to be repeated over time might be different for each type of injury, the adoption of biomechanical adjustments by runners to protect themselves from a previous injury might contribute to the recurrence of that injury [154]. Therefore, assuring complete recovery from a previous injury and using a gradual increase in training load in the first weeks of training after recovery could be crucial to avoiding injury recurrence [155].

Overall, the most common body location for lower extremity injuries in recreational endurance runners is the knee [148], with the existence of some investigations indicating that knee injuries might represent half of all injuries in recreational runners [145]. Although the lower leg, the foot, and the thigh are other common body locations for running-related injuries, the frequency of injury in these body locations is lower than 40% [156,157]. Interestingly, the location of injury was indicated to vary depending on the training mileage and experience; the most common location in novice runners is the lower leg, but the knee is most common in more experienced runners [148]. Endurance runners tend to suffer progressive-onset injuries, which are catalogued as overuse injuries [158]. Among them, medial tibial stress syndrome, Achilles tendinopathy, anterior knee pain, and plantar fasciitis are the most common [156,158]. A number of other injuries and syndromes can be found in recreational runners as the result of their training practice or competitive activity with different anatomical and biomechanical explanations [159]. Repetitive overloading seems to be the most probable mechanistic cause for most injuries. For this reason, runners who develop stride patterns with low levels of impact force are at a reduced risk of incurring overuse running injuries [160]. In addition, longer recovery periods should be encouraged to assure adaptation and recovery between training sessions, as these might be important to reduce the risk of running-related injuries. 

As mentioned above, it seems that a high running mileage per week is the most important injury risk factor among recreational endurance runners; for this reason, training for races of greater distance (e.g., ultra-endurance competitions) is also a risk factor for incurring running-related injuries [148,149]. Other training aspects, such as running pace and surface, were found to be contributing factors of risk injury, although their contributions are less than training mileage [145,161]. Interestingly, age does not seem to produce a protective or negative role for the development of injury [145]; thus, both younger and older runners might be similarly exposed to injury and the probability of sustaining an injury mainly depends on other factors, such as previous injury and running mileage per week. However, the influence of running mileage per week on the risk of injury should not be interpreted as a clear and direct effect of the training volume on the likelihood of injury, as all publications show that a considerable proportion of runners can undergo high volumes of training without any signs of injury. In this regard, a high running volume per week might be a “trigger” factor that exacerbates the effect of other risk factors for running-related injuries, subsequently increasing the overall probability of injury. However, if training is balanced according to the characteristics and experience of the athlete while avoiding both overuse and underconditioning, injury risk is expected to be controlled even in the case of conducting high volumes of training. 

Women seem to be at lower risk than men for sustaining running-related injuries [162]. Interestingly, male runners are more prone to injuries located in the hamstring and calf, while women are more prone injuries of the hip [163]. In addition, high volumes of running on asphalt roads and wearing the same running shoes for four to six months was associated with a greater risk of injury in women [162] but not in men. Importantly, irregular or absent menstruation was associated with an increased risk of bone injuries [153]. However, sex-related differences are subtle and do not imply the use of different recommendations for males and females.

It is a common belief that shoe cushioning technology protects against running-related injuries, but current evidence does not provide any support for the beneficial effect of increased shock absorption to reduce the likelihood of injury [164,165]. Recent evidence suggested that optimal shoe cushioning properties might differ depending on the athlete’s body mass, because greater shoe cushioning might only benefit lighter runners [166].

The use of stretching exercises has become a key part of athletic training for endurance runners because of athletes’ perceptions that it prevents injury and may enhance running performance [167]. However, the evidence is contradictory regarding the assumption that better muscle flexibility, especially in the lower extremities, reduces the risk of injury in endurance runners [168]. Muscle flexibility was suggested to neither improve nor decrease likelihood of endurance running injuries, although extreme cases outside the normal range of flexibility might be problematic because of interference with the normal pattern of running [169]. In addition, the use of chronic stretching is effective to increase muscle flexibility but might negatively impact endurance running economy [20]; therefore, stretching could be performed to allow normal range of mobility during running.

The available evidence is insufficient to determine whether total energy intake per day or macronutrient and micronutrient proportions in the diet are associated with the prevalence of injury in endurance runners [170]. Further investigations are needed to determine whether caloric restriction is related to a higher incidence of injury, as diets with reduced calorie intake are gaining popularity [171] and no evidence exists on this topic. Finally, several dietary supplements are consumed by athletes to alleviate pain and to reduce the risk of injury based on the claims of supplement manufacturers. However, the recent consensus statement by the International Olympic Committee indicated that only the use of vitamin D to treat nutrient deficiencies that can lead to bone injuries and the use of creatine supplementation to maintain lean mass during periods of immobilization after injury are sufficiently supported by science [172].

In summary, the evidence indicates that several intrinsic (sex, running biomechanics, anatomy) and extrinsic factors (experience, mileage, training routines) might contribute to the risk of injury in recreational runners. While the history of a previous injury of the same type and in the same location is an intrinsic and unmodifiable factor with great influence on risk of injury, other factors, such as appropriate running volume and intensity, especially in novice runners, and the use of adequate running shoes, might contribute to reducing the likelihood of injury. Special attention should be paid to preparation for long-distance events, such as marathons and ultra-endurance races, because running mileage should be balanced to ensure conditioning without increasing the risk of overuse injuries. Runners with one or more risk factors for endurance running injuries should be more aware of the first signs of injury and should immediately change their training practices or cease training to elude other risk factors.

## 7. Conclusions

**Figure 1:**
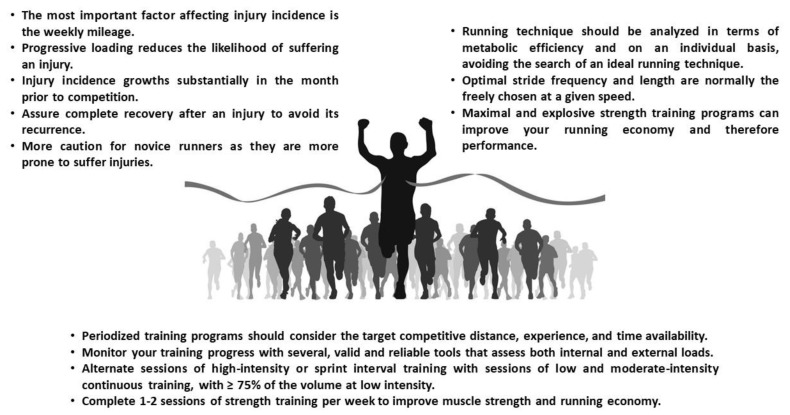
Evidence-based recommendations for physical conditioning training, running technique, and injury prevention in recreational endurance running.

In this narrative review, we have presented a balanced overview, based on scientific literature, of some key aspects of endurance running training for recreational athletes. We focused the review on training methodologies, periodization, training monitoring, performance prediction, running technique, and management and prevention of running-related injuries. While we acknowledge that some information may be missing because of the broad scope of this work, the current review may be useful and practical for coaches to better manage the training process of recreational runners of all performance levels. This information is summarized in Figure 1. Further studies should be conducted to specifically address the identified gaps of literature that are currently used in practice but without enough scientific support.
